# Derivative chromosome 1 and *GLUT1 *deficiency syndrome in a sibling pair

**DOI:** 10.1186/1755-8166-3-10

**Published:** 2010-05-28

**Authors:** Dilek Aktas, Eda G Utine, Kristin Mrasek, Anja Weise, Ferdinand von Eggeling, Kalbiye Yalaz, Nicole Posorski, Nurten Akarsu, Mehmet Alikasifoglu, Thomas Liehr, Ergul Tuncbilek

**Affiliations:** 1 Department of Genetics, Hacettepe University School of Medicine, Ankara, Turkey; 2 University Hospital, Institute of Human Genetics, Jena, Germany; 3 Department of Pediatric Neurology, Hacettepe University School of Medicine, Ankara, Turkey

## Abstract

**Background:**

Genomic imbalances constitute a major cause of congenital and developmental abnormalities. GLUT1 deficiency syndrome is caused by various de novo mutations in the facilitated human glucose transporter 1 gene (1p34.2) and patients with this syndrome have been diagnosed with hypoglycorrhachia, mental and developmental delay, microcephaly and seizures. Furthermore, 1q terminal deletions have been submitted in the recent reports and the absence of corpus callosum has been related to the deletion between *C1orf100 *and *C1orf121 *in 1q44.

**Results:**

This study reports on a sibling pair with developmental delay, mental retardation, microcephaly, hypotonia, epilepsy, facial dysmorphism, ataxia and impaired speech. Chromosome analysis revealed a derivative chromosome 1 in both patients. FISH and MCB analysis showed two interstitial deletions at 1p34.2 and 1q44. SNP array and array-CGH analysis also determined the sizes of deletions detailed. The deleted region on 1p34.2 encompasses 33 genes, among which is *GLUT1 *gene (*SLC2A1*). However, the deleted region on 1q44 includes 59 genes and distal-proximal breakpoints were located in the ZNF672 gene and SMYD3 gene, respectively.

**Conclusion:**

Haploinsufficiency of *GLUT1 *leads to GLUT1 deficiency syndrome, consistent with the phenotype in patients of this study. Conversely, in the deleted region on 1q44, none of the genes are related to findings in these patients. Additionally, the results confirm previous reports on that corpus callosal development may depend on the critical gene(s) lying in 1q44 proximal to the *SMYD3 *gene.

## Background

Mental retardation (MR) affects around 3% of the population [1,2]. Genomic imbalances constitute a major cause of congenital and developmental abnormality. Chromosomal abnormalities are reported in 30-40% of patients with moderate/severe MR and in 10-15% of patients with mild MR [3], while submicroscopic subtelomeric chromosomal rearrangements may account for as high as 6.8-7.4% of patients with moderate-severe MR and for 0.5-1.1% of patients with mild MR [3].

Chromosomal changes cause phenotypic findings depending on the size and content of the genomic imbalance. Current data on deletions of 1p34.3p34.1 indicate that these cause minor anomalies, hyperactivity and severe developmental delay [4,5]. *GLUT1 *gene located in this region has recently appeared to cause a specific syndrome with drug-resistant epilepsy, developmental delay, microcephaly, spasticity, ataxia [5].

Microscopically visible terminal deletions of 1q lead to a recognizable phenotype with mental retardation, microcephaly, growth retardation and characteristic facial features [6]. Various midline abnormalities, particularly absence of corpus callosum, were reported in 1qter deletions [6]. Recent reports suggested that absence of corpus callosum was related to deletions between the genes *C1orf100 *and *C1orf121 *in 1q44 [7].

Routine cytogenetics detects chromosomal imbalances of at least 3-5 Mb in size, limited to resolution of GTG-banding at 450-600 bands [8]. In the last two decades, traditional banding has been combined with targeted molecular technologies in order to improve the resolution of cytogenetic techniques. Precise delineation of submicroscopic rearrangements may allow identification of responsible genes for some clinical features [7]. Array-CGH and SNP oligonucleotide array analysis allows even more precise delineation of the genomic imbalances and genes that possibly contribute to the clinical findings of the patients may be identified.

We present clinical findings of a sibling pair with 1p34.2 and 1q44 deletions. The data are compared with previously reported patients having similar chromosomal abnormalities and genes that possibly contribute to the clinical findings of these patients are discussed.

## Clinical Presentation

### Case Reports

#### Case A

The patient is a 10.5-year-old son of healthy nonconsanguineous parents. He was born at 38 weeks of gestational age with a birth weight of 1950 g. His development was normal until 10 months of age; however, there was delay in sitting without support until 1.5 years of age. He walked after 2 years of age and he never talked. Epilepsy was diagnosed at 7 years of age. He received special education for three years, but his mental status never improved.

**Figure 1:**
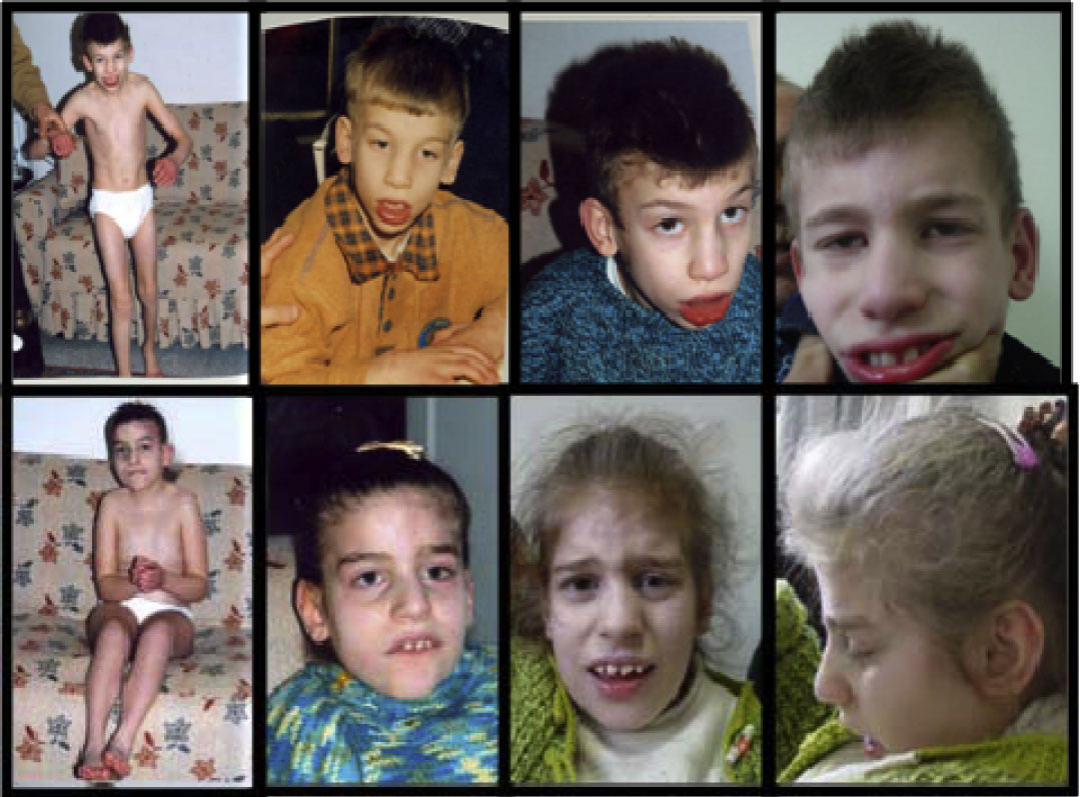
**Facial appearance of the patients**. Note the deeply-set eyes, large mouth with full lips and widely spaced teeth, and prognathism.

On physical examination, weight (24 kg) and height (126 cm) were both between 3rd-10th centiles, and head circumference (48.5 cm) was below 3rd centile. He had deeply-set eyes, large mouth with full lips and widely spaced teeth, and prognathism (Figure 1). He had ataxia and was unable to walk independently. Clinical features of patients are summarized in Table 1.

**Table 1:** Clinical findings of patients with 1q deletion.

Feature	Present patients	van Bon et al., 2008	van Bever et al., 2005	de Vries et al., 2001
**Karyotype**	**Monosomy 1q44→1qter**	**Monosomy 1q43→q44**	**Monosomy 1q43→q44**	**Monosomy 1q43→qter**

Low birth weight	+/-	8/11	+	+

Microcephaly	+/+	11/11	+	+

Short stature	-/-	10/11	+	+

Hypotonia	+/+	10/11	?	+

Mental retardation	+/+	11/11	+	+

Impaired speech	+/+	10/11	+	+

Motor delay	+/+	10/11	+	+

Epilepsy	+/+	9/11	+	+

Deep set eyes	+/+	10/11	+	+

Widely spaced teeth	+/+	8/11	+	?

Thin bow shaped upper lip	+/+	11/11	+	+

Abnormality of corpus callosum	-/-	9/11	+	+

Scoliosis	-/+	2/11	+	+

Ataxia	+/+	0/11	-	-

**Figure 2:**
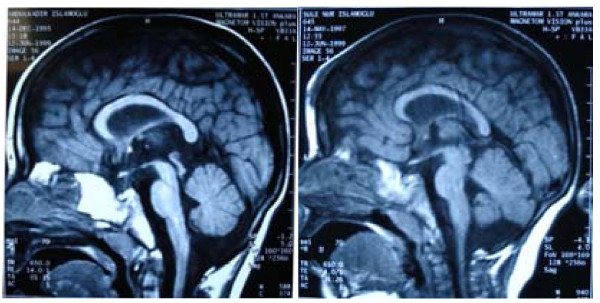
**Cranial MRI showing diffuse cerebral atrophy with a relatively normal sized corpus callosum, relatively large lateral ventricules and normal sized brain stem and cerebellum**.

Stanford-Binet scale of intelligence, performed at 4 years and three months of age, revealed an IQ of 36. A previous cranial MRI revealed diffuse cerebral atrophy with a relatively normal sized corpus callosum, relatively large lateral ventricles and normal sized brain stem and cerebellum (Figure 2). Ophthalmologic and cardiac examinations were normal.

As GLUT1 deficiency syndrome was suspected after completion of the SNP oligonucleotide array analyses, lumbar puncture was performed following a period of 6 hours fasting, to detect the presence of hypoglycorrachia. Blood glucose was 83 mg/dl (70-110), while simultaneous CSF glucose was 43 mg/dl (40-80). CSF lactate was 16.2 mg/dl (9.91-20.72) and no cells were present in the CSF. CSF/blood glucose ratio was calculated as 0.52, and considering the clinical picture, was acceptable as hypoglycorrachia. Ketogenic diet was initiated.

#### Case B

The patient was the 9-year-old sister of the first patient who was born at term with a birth weight of 3000 g. Her development was normal until 10 months of age; however, there was a delay in sitting without support until 1 years of age. She walked at three years of age and she never talked. She had her first convulsive attack at the age of three months and epilepsy was diagnosed at the age of six years. She had special education since 5 years of age with no improvement at all. She had difficulty in swallowing and she had ataxia.

On physical examination, she weighed 26 kg (25th-50th centiles). Her height was 120 cm (3rd-10th centiles) and her head circumference was 50.5 cm (25th centile). She had deeply set eyes, large mouth, full lips, widely spaced teeth and prognathism (Figure 1). She had ataxia and was unable to walk independently.

Denver Developmental Screening Test was performed at the age of 2 years and 8 months, which revealed delay in all four categories. A previous cranial MRI revealed diffusely atrophic brain with a relatively normal sized corpus callosum, relatively large lateral ventricles and normal sized brain stem and cerebellum. Overall, cerebral atrophy was more severe than in her brother. Ophthalmologic and cardiac examinations were normal. Lumbar puncture for detection of hypoglycorrachia could not be performed because of the presence of scoliosis.

Written informed consents were obtained from the patient's parents for publication of these cases reports and accompanying images. A copy of the written consent is available for review by the Editor-in-Chief of this journal.

## Methods

Cytogenetic analyses were performed on GTG-banded metaphase spreads prepared from phytohemagglutinine (PHA)-stimulated peripheral blood lymphocytes and chromosome preparation techniques. The karyotypes were described in accordance with ISCN [9]. Chromosome analyses were done in 50 metaphases for each sample with a resolution of 550 bands.

Whole chromosome painting for chromosome 1 was done using commercially available probes (Vysis^®^, Abbott Laboratories) according to manufacturer's instructions. Subtelomeric FISH analyses for 1q were done applying the corresponding commercially available probes (Vysis^®^, Abbott Laboratories). Multicolor chromosome banding (MCB) for chromosome 1 was done as described previously [10].

Genomic DNA of 250 ng from a sample of previously isolated genomic DNA was used. Affymetrix 500 K Assay Protocol and GeneChip^® ^Human Mapping 250 K Nsp Arrays were used for Genotyping (Affymetrix Inc. Santa Clara CA, USA) at Hacettepe University School of Medicine. QC analysis were performed using Affymetrix's software GCOS (Gene Chip Operating Software). Genotyping Console V2.0 was used for further Copy Number Variation (CNV) analysis. 50 HapMap 250 K samples were randomly selected from HapMap Data Base and used to create a Reference Set for CNV analysis. Furthermore, CNV analysis were then confirmed using Partek^® ^Genomics Suite v6.3 and CNV regions were also checked with Genotyping Console and Toronto Database to determine which genes are involved in those regions.

Array-CGH analysis was performed according to the manufacturere's protocol (Agilent, Technologies, Santa Clara, CA). The investigation was made using a female genomic DNA pool as reference. The quality of the experiment was evaluated with the QC metric provided by the CGH analysis software (CGH Analytics 3.5.14, Agilent Technologies).

## Results

**Figure 3:**
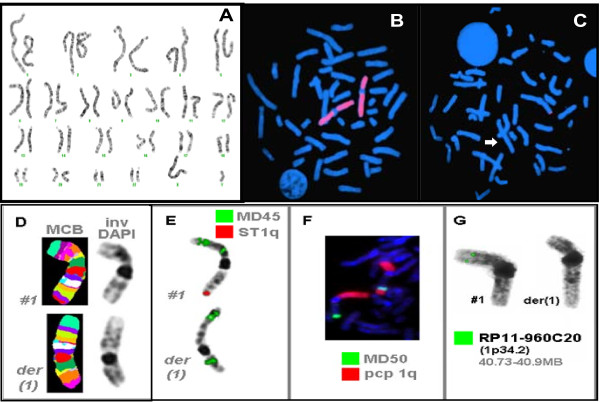
**Karyotype of patients showing in (A), FISH with whole chromosome paint 1 exclude the posibility of the missing material elsewhere in the genome (B) and subtelomeric FISH presenting in (C)**. Multicolor chromosome banding for Patient 1 clearly shows an extremely altered pattern on the derivative chromosome 1 (D). Using additional microdissection derived probes (MD45: stains 1pter-1p36.2, 1p35-1p33 and 1p22, MD50: stains 1q42-qter) in combination with a subtelomeric probe for chromosome 1qter (ST1q) or a partial chromosome paint for the long arm of chromosome 1 (pcp 1q) are shown in figure parts E and F, respectively (E-F). To control the results of array-CGH BAC RP11-960C20 in 1p34.2 was applied and confirmed deletion of the corresponding region on the derivatve chromosome 1. Thus, the derivative chromosome 1 could be described as der(1)(pter->p34.2::q43~44->p34.2:) (G).

GTG-banded cytogenetic analysis at 550 band resolution revealed a derivative chromosome 1. The karyotypes of the siblings were described as 46,XY, der(1)(pter->p34.2::q43~44->p34.2:) and 46,XX, der(1)(pter->p34.2::q43~44->p34.2:) (Figure 3). FISH analysis using WCP probe excluded the possibility of a translocated material elsewhere in the genome. Using subtelomeric probes, FISH analysis for chromosome 1 showed the deletion on 1q44. Furthermore, MCB analysis [11] demonstrated the presence of deletion at 1p34.2 and 1q43~44 (Figure 3).

Cytogenetic studies from the parents showed that the paternal karyotype was normal. However, the maternal karyotype was similar. Subtelomeric FISH analysis showed the deletion on 1q44 in healthy women. But, no deletion on 1p34.2 was found using array-CGH and SNP array analysis in this mother.

**Figure 4:**
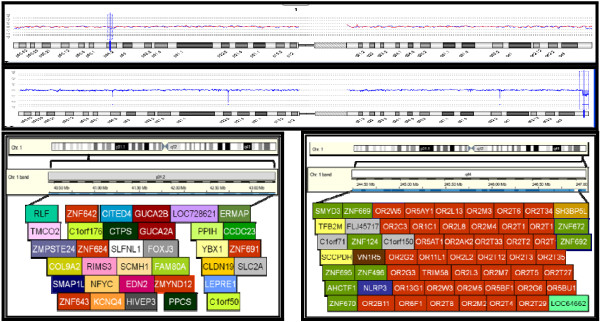
**Array-CGH analysis presents the deletion on 1p34.2 and 1q44**. Schematic representation showing the known genes with the deleted region. An ideogram of chromosome 1 is shown on top. The region of the short arm from band 1p34.2 (on the left) and the region of the long arm from band 1q44 (on the right) are enlarged below. The names of known genes and their relative functions are indicated by the different colors http://www.ensembl.org/index.html.

Using SNP array, an interstitial deletion of 2.9 Mb in segment 1p34.2 (40430036-43332174 bp) and 2.7 Mb in segment 1q44 (244444664-247110269 bp) were detected in patients. Additionally, array-CGH analysis was also performed and deletions in 1p34.2 and 1q44 were presented. In these regions, a number of deleted genes were observed http://www.ensembl.org/index.html as shown in Figure 4. At 1p34.2, distal breakpoint was located in the *RLF *gene and proximal breakpoint was in *SLC2A1 *gene. However, at 1q44, distal breakpoint of 1q44 was located in *ZNF672 *gene and proximal breakpoint was located in *SMYD3 *gene (Figure 4).

## Discussion

The present sibling pair with interstitial deletions of 1p34.2 and 1q44 supports two previous findings on 1q and 1p deletions. First, we confirm that haploinsufficiency of *GLUT1 *may lead to GLUT1 deficiency syndrome in interstitial deletions of 1p34.2, which was previously described in patients with *GLUT1 *mutations [12]. Second, our findings support that absence of corpus callosum is related to deletions of a short segment of 300 kb between the genes *C1orf100 *and *C1orf121 *in 1q44 [7].

Our data indicates that an interstitial deletion of 1p34.2 must be ruled out in patients presenting with a clinical picture of *GLUT1 *deficiency syndrome, which is characterized by epilepsy, developmental delay, microcephaly, hypotonia, ataxia and impaired speech. In the presented patients, the deletion in 1p34.2 required high-resolution analysis to visualize the abnormality. With increasing use of SNP-array in the clinical area, descriptions of additional patients will allow more precise syndrome delineation.

To date, only twenty-three submicroscopic 1q terminal deletions have been submitted of which nine were interstitial [7,13-15]. Our findings show that absence of corpus callosum is not a clinical finding in deletions involving the region distal to *SMYD3 *gene, confirming the previous findings [7]. Further identification of the four genes lying in this region, *C1orf100, ADSS, C1orf101 *and *C1orf121 *and delineation of their functions will clarify the unknown mechanisms in formation of corpus callosum.

Further examinations of the genes at 1p34.2 and at 1q44 are likely to reveal dosage-sensitive genes that may contribute to many of these phenotypes. In the presented patients, 33 genes are located in the deleted region at 1p34.2 (Figure 4), of which 19 have been previously described in OMIM database. So far, only seven (*ZMPSTE24, COL9A2, LEPRE1, KCNQ4, CLDN19, SCL2A1*) have been associated with well-known disorders (see Table S1; Additional File 1). Among these, *GLUT1 *(*SCL2A1*) gene seemed to be related to the phenotype of our siblings. On the other hand, 59 genes are located in the deleted region at 1q44 (Figure 4), of which 5 are described in OMIM database and only two (*SMYD3*, *OR13G1*) have been associated with a disease (see Table S1; Additional File 1). Based on the presented data of patients and healthy mother, a deletion at 1q44 was not contribute to clinical phenotypes and we assume that haploinsufficiency of *GLUT1 *was responsible for the neurological findings of our patients.

## Conclusion

We conclude that molecular characterization of genomic imbalances in patients with developmental retardation will clarify many unknown aspects of human development, as suggested by the already accumulated data. Haploinsufficiency of *GLUT1 *due to deletions of 1p34.2 leads to GLUT1 deficiency syndrome which may be a clinically recognizable condition in dysmorphic patients. Corpus callosal development seems to be very much dependent on the critical gene(s) lying in a short segment of 300 kb between the genes *C1orf100 *and *C1orf121 *in 1q44, and not distal to this region.

## Competing interests

The authors declare that they have no competing interests.

## Authors' contributions

DA: carried out cytogenetic studies, SNP-array analysis and drafted the manuscript; EGU: carried out clinical examination and evaluation; KM: carried out molecular genetic studies; AW: carried out MCB analysis; FVE: carried out molecular evaluation; KY: carried out neurological examination; NP: carried out array-CGH analysis; NA: carried out molecular evaluation; MA: carried out molecular evaluation and SNP-array analysis; ET: carried out clinical evaluation. All authors read and approved the final manuscript.

## Supplementary Material

Additional file 1 **Table S1**. The results of SNP array analysis. Click here for file
