# Supplementary material for: Derivative chromosome 1 and GLUT1 deficiency syndrome in a sibling pair
Source: Mol Cytogenet. 2010 May 28;3:10. doi: 10.1186/1755-8166-3-10 (PMC2887874; doi:10.1186/1755-8166-3-10)
Supplement: Additional file 1 — Table S1. The results of SNP array analysis. [file 1755-8166-3-10-S1.DOC]

**Additional file1, Table S1
The results of SNP array analysis**

**Gene Location OMIM Related Disease References**

***ZMPSTE24***  1p34 606480 Mandibular dysplasia Hum Molec Genet 2003;12:1995-2001

(zinc metallopeptidase ([608612](http://www.ncbi.nlm.nih.gov/entrez/dispomim.cgi?id=608612))

(STE24 homolog)

***COL9A2*** 1p33-p32 120260 Epiphyseal dysplasia Am. J. Med. Genet 2002;112:144-153,

(collagen, type IX, ([600204](http://www.ncbi.nlm.nih.gov/entrez/dispomim.cgi?id=600204))

alpha 2)

***KCNQ4***  1p34 603537 Autosomal dominant J. Hum. Gene 2006; 51: 455-460

(potassium voltage-gated non-syndromic sensoro-

channel, KQT-like sub- neural deafness type 2

family member 4) ([600101](http://www.ncbi.nlm.nih.gov/entrez/dispomim.cgi?id=600101))

***LEPRE1*** 1p34.1 610339 Osteogenesis imperfecta New Eng J Med 2006;355:2757-2764

(leucine proline-enriched type VIII (610915)

proteoglycan (leprecan) 1)

***CLDN19*** 1p34.2 610036 Hypomagnesium with Am J Hum Genet 2006;*79:949-957*

(Claudin 19) renal failure and ocular invol-

vement ([248190](http://www.ncbi.nlm.nih.gov/entrez/dispomim.cgi?id=248190)),

***SCL2A1 (GLUT1)*** 1p34.2 138140 Noninsulin-dependent Ann. Neurol. *50: 476-485, 2001.*

solute carrier family 2 diabetes mellitus (NIDDM) New Eng J Med 1991; 325: 703-709

(facilitated glucose trans- ([125853](http://www.ncbi.nlm.nih.gov/entrez/dispomim.cgi?id=125853)) and epilepsy

porter), member 1

***SMYD3***  1q44 608783 Colorectal and hepatocel- Nature Cell Biol 2004;*6:* 731-740

(SET and MYND lular carcinomas

domain containing 3)

***OR13G1*** 1q44 611677 Myocardial infarction Proc Nat Acad Sci 2004;101:2584-2589

(olfactory receptor,

family 13, subfamily G,

member 1)

***C6orf66*** 6q16.3 611776 Infantile mitochondrial Am J Hum Genet; 2008:82:33-38

(chromosome 6 open encephalopathy

reading frame 66) (252010)

***SIM1*** 6q16.3-q21 603128 Hyperphagia, obesity Hum Mol Genet; 2001:10:1465-73

(single-minded homo- and reduction of the para- J Med Genet; 2002:39:594-596

log 1) ventricular nucleus of the

hypothalamus

***GRIK2 (GLUR6)*** 6q16.3-q21 138244 Autosomal recessive mental Am J Hum Genet 2007;81:792-798

(Glutamate receptor, iono- retardation and relation with Proc. Nat. Acad. Sci 1997;94:3872-76

tropic, kainate 2) Hungtington disease

***HACE1***  6q21 610876 Wilms tumor Hum Molec Genet 2004;13:2061-74

(HECT domain and ankyrin

repeat containing, E3 ubi-

quitin protein ligase 1)

***AIM1***  6q21 601797 Malign melanoma Proc. Nat. Acad. Sci. 1997;94:3229-34

(Absent in melanoma 1)

***PDSS2***  6q21 610564 Leigh syndrome with Am J Hum Genet 2006;79:1125-29

(Prenyl diphosphate nephropathy and CoQ10

Synthase, subunit 2) deficiency

***SEC63***  6q21 608648 Autosomal dominant Nat Genet 2004;36:575-576

(SEC63 homology) polycystic liver disease

***OSTM1***  6q21 607649 Autosomal recessive J Bone Miner Res 2006;21:1098-1105

(Osteopetrosis assocaited osteopetrosis

transmembrane protein 1)

***SNX3***  6q21 605930 Microcephaly, micropt- J Med Genet 2002;39:893-899

(Sorting nexin 3) halmia, ectrodactyly and

Prognathism
